# 

**Published:** 1893-02-04

**Authors:** 


					^ Vol. XIII.- Feb. 4th, 1893 M, f)
tt*&*aii?.L-e General Poat Office as a Newspaper or ST>-/
on in the United Kingdom and Abroad.]
uui
?o,^jl(?E TWOPENCE,.. fell ,, 2LH5Z2?
332. Vol. XIII.] Saturday,
February 4th, 1593.
[Twopence.

				

